# Discrimination and Psychological Distress: Gender Differences among Arab Americans

**DOI:** 10.3389/fpsyt.2017.00023

**Published:** 2017-02-20

**Authors:** Shervin Assari, Maryam Moghani Lankarani

**Affiliations:** ^1^Department of Psychiatry, School of Medicine, University of Michigan, Ann Arbor, MI, USA; ^2^Center for Research on Ethnicity, Culture and Health, School of Public Health, University of Michigan, Ann Arbor, MI, USA; ^3^Medicine and Health Promotion Institute, Tehran, Iran

**Keywords:** Arab Americans, gender, discrimination, psychological symptoms, distress

## Abstract

**Background:**

Despite the existing knowledge on the association between discrimination and poor mental health, very few studies have explored gender differences in this association in Arab Americans.

**Objective:**

The current study aimed to investigate whether gender moderates the association between the experience of discrimination and psychological distress in a representative sample of Arab Americans in Michigan.

**Methods:**

Using data from the Detroit Arab American Study (DAAS), 2003, this study recruited Arab Americans (337 males, 385 females) living in Michigan, United States. The main independent variable was discrimination. The main outcome was psychological distress. Covariates included demographic factors (age), socioeconomic status (education, employment, and income), and immigration characteristics (nativity and years living in United States). Gender was the focal moderator. We used multivariable regression with and without discrimination × gender interaction term.

**Results:**

In the pooled sample, discrimination was positively associated with psychological distress [*B* = 0.62, 95% confidence interval (CI) = 0.22–1.03, *p* = 0.003]. We found a significant gender × discrimination interaction in the pooled sample (*B* = 0.79, 95% CI = 0.01–1.59, *p* = 0.050), suggesting a stronger association in males than females. In our gender-specific model, higher discrimination was associated with higher psychological distress among male (*B* = 0.87, 95% CI = 0.33–1.42, *p* = 0.002) but not female (*B* = 0.18, 95% CI = −0.43 to 0.78, *p* = 0.567) Arab Americans.

**Conclusion:**

While discrimination is associated with poor mental health, a stronger link between discrimination and psychological symptoms may exist in male compared to female Arab Americans. While efforts should be made to universally reduce discrimination, screening for discrimination may be a more salient component of mental health care for male than female Arab Americans.

## Introduction

Discrimination can be defined as the unfair treatment of different categories of people based on race, gender, age, religion, sexual orientation, and other characteristics (1–4). Given that discrimination operates as a stressor, it may increase the risk of a wide range of undesired physical and mental health outcomes (5–9). Some of the undesired consequences of discrimination include depression (10), anxiety (11), substance use (12), suicide (13, 14), and psychological distress (15).

From a population perspective, most of the United States literature on the association between the discrimination and mental health of racial and ethnic minorities is written about discrimination against Blacks (3, 8, 16–22) and Hispanics (23–25). Thus, more is yet to be learned about populations such as Arab Americans (26, 27) who also experience considerable levels of discrimination in their everyday life (28).

Regarding causes of discrimination, most of the current literature is written about discrimination because of race and ethnicity; however, religion is also a source of discrimination (13, 14). This is especially important for Muslim Americans, a population commonly exposed to discrimination for reasons other than race and ethnicity, specifically their belief in Islam (26–28). Particularly, discrimination against the Muslim American community increased considerably following September 11 attacks (29, 30).

Regardless of whether it is overt or perceived, general or specific, related to race, ethnicity, race, or religion, exposure to discrimination is a risk factor for poor mental health (3, 9, 16, 31–35). Perceived discrimination influences may have long-term effects on mental health (31). This notion proposes perceived racial discrimination as a distinct stressor (31). Perceived discrimination can lead to a wide range of adverse mental outcomes including depression (10), anxiety (11), substance use (12), suicidal ideation (13, 14), and psychological distress (15).

Discrimination may have similar effects as other stressors (3). There are studies that suggest discrimination may even have a more powerful effect on psychological distress compared with general stressful life events (9, 36). Baseline perceived discrimination better predicts future adverse mental health outcomes than *vice versa*, suggesting a causal link between discrimination and poor mental health (16).

From a long list of potential moderators that alter the effects of discrimination on mental health (10, 37, 38), gender has received little attention (12). Gender interferes with experiencing discrimination (39–42). For instance, among Blacks, men report higher rates of perceived discrimination compared to women (43, 44). Concerning the gender gap in discrimination, multiple hypotheses are proposed. The race–gender intersectionality hypothesis suggests that life experiences are shaped by the intersection of race and gender, rather than merely race or gender alone, and discrimination may be more common among Black men (41). This is also in line with mass incarceration and police brutality against Black men (45, 46). Based on the subordinate male hypothesis, discrimination is more detrimental for men than women, as men have a higher preference for dominance and hierarchy. In this view, masculinity ideologies may increase mental health costs associated with perceived discrimination (47).

The current study was performed to investigate whether gender moderates the association between discrimination and psychological distress in a representative sample of Arab Americans in the United States.

## Materials and Methods

### Design and Setting

Data came from the 2003 Detroit Arab American Study (DAAS) survey (48). The DAAS is a representative survey conducted from July to December 2003 in Wayne, Oakland, and Macomb counties, Michigan. The DAAS survey was designed through a partnership and collaboration between the academic affiliates and community members where both Muslim Arab and Chaldean (Christian) Arab groups were represented.

### Main Study

The DAAS data collection was conducted between July and December 2003. The DAAS included all adults of Arab or Chaldean descent living in Michigan’s Wayne, Oakland, and Macomb counties. Approximately 490,000 Arabs resided in Michigan at the time of study, of whom more than 80% lived in the above listed counties (49). Arab Americans are the third largest ethnic group in Michigan, with a history dating back multiple generations (30, 50, 51).

Some of this population are Christian, and some are Muslim. For instance, Chaldean Americans are Catholic Christians and are descendants of people from the northern Tigris-Euphrates Valley, with their own language. Although they share some aspects of culture and experience with other Arab Americans, they may also represent a distinct ethnic group (52, 53). Thus, although participants in this study represent distinct ethnic groups, they share major aspects of Arab cultures and are all recognized as Arab Americans (30).

### Sampling

Eligibility was based on Arab or Chaldean ancestry from adults living in Michigan. Most countries included were Egypt, Iraq, Jordan, Palestine, Lebanon, Libya, Saudi Arabia, Syria, and Yemen. Although these countries are all Arab, they are not all Muslim countries. A large proportion of participants were also Chaldean Christians. Individuals residing in institutions, in group quarters, or on military bases were excluded (54). A total of 1,389 eligible households were identified; 1,016 adults from these eligible households completed the study interview. Participation rate was 73% (30).

A dual-frame probability sample design was used in DAAS. An area probability frame was employed to select area segments from year 2000 census tracts in which 10% or more of individuals self-identified as Arab or Chaldean in ancestry. Then a list frame was used to select housing units from the mailing and membership lists of 13 Arab American and Chaldean American community organizations. The area probability component was based on the following stages: (1) area segment units, (2) housing units within area segments, and (3) selection of an eligible adult from each household. Within the list frame, a systematic random sample of individual addresses was employed, with random selection of one eligible adult respondent in each household (30).

### Process

The DAAS administered surveys *via* face-to-face interviews for data collection. Data were collected on demographic and socioeconomic factors, discrimination, attitudes and beliefs, and health. The main predictor of interest was discrimination. The main outcome was psychological distress. Confounders included demographic and socioeconomic factors and health status (30).

### Measures

#### Demographic Factors

Data were collected on gender (male as the referent group) and age (younger than 30 years, 30–55 years, older than 55 years).

#### Socioeconomic Characteristics

We collected data on education level (less than high school, some college, and advanced degree), marital status, and household income (less than $30,000, $30,000–$75,000, and above $75,000).

#### Immigration-Related Factors

Data were collected on the country of origin, nativity, and years of residence in the United States.

#### Discrimination

The study collected data on self-report of personal abuse against individuals or their household members by answering yes or no to the question “*In the last 2 years, have you personally, or anyone in your household, experienced verbal insults or abuse, threatening words or gestures, physical attack, vandalism or destruction of property, or loss of employment, due to your race, ethnicity, or religion?*” Participants were also asked if they had experienced post-September 11 discrimination by the following question “*Since 9/11, have you personally had a bad experience due to your Arab or Chaldean ethnicity?*” Respondents were also asked to rate their level of agreement, on a 5-point Likert-type scale, with the statement “*Arab Americans are not respected by the broader American society*.” Fourth, they were asked “*How much—if any—have the events of 9/11 shaken your own personal sense of safety and security?*” Responses were gathered on a 4-point Likert-type scale.

#### Self-rated Health (SRH)

To measure SRH, all respondents were asked “*How would you describe your overall state of health these days? Would you say it is excellent, very good, good, fair, or poor?*” This single-item measure of SRH has been shown to be an independent predictor of mortality risk above and beyond baseline physical health status and lifestyle (55, 56).

#### Psychological Distress

The main outcome variable was psychological distress measured by the Kessler Psychological Distress Scale (K10) (57). The K10 results have been previously validated as a screening tool comparable with the General Health Questionnaire (58). The Cronbach alpha coefficient for the K10 in our sample was 0.89. We computed a K10 score for each respondent, with a higher score reflecting more distress.

### Data Analysis

Data analysis was conducted in SPSS 20.0 for Windows (IBM Inc., Armonk, NY, USA). For descriptive statistics, we presented frequency and relative frequency for categorical and mean measures, and SD for continuous measures, both in the pooled sample, and based on gender. We used the Pearson correlation test for bivariate analysis, in the pooled sample, and based on gender.

For our multivariable analysis, we ran a series of multiple linear regressions. First, we ran our models in the pooled sample. In these models, discrimination was the main independent variable of interest, and psychological distress was the dependent variable. Age, education, income, marital status, employment, and SRH were covariates. Gender was the focal moderator. Regression coefficients (*B*), SE, 95% confidence interval (CI), and *p* value were reported.

In the first regression model, we only included demographic factors (age, gender, and nativity). In model 2, we also added socioeconomic factors (education, family income, married, and employment). In model 3, we also controlled for health (SRH) added discrimination and included gender by discriminate interaction. Then we ran gender-specific models with the same order (models 1–4).

### Ethics

The research followed the tenets of the Declaration of Helsinki. This project was approved by the Institutional Review Board of the University of Michigan, Ann Arbor. All participants provided written informed consent, and all data were kept confidential.

## Results

### Univariate Analysis

Table 1 shows descriptive statistics in the pooled sample. As this table shows, mean (SD) of age of the participants was 43.63 (16.48). Most of the participants were women, immigrants (non-United States born), married, and unemployed.

**Table 1:** **Summary of descriptive statistics in the pooled sample and based on gender in Arab Americans**.

	All		Men		Women	
	*n*	%	*n*	%	*n*	%
Gender						
Men	538	53.6	–	–	538	100
Women	466	46.4	466	100	–	–
Nativity						
Foreign born	732	72.4	337	72.8	385	71.8
United States born	279	27.6	126	27.2	151	28.2
Married						
No	292	28.7	126	27.0	164	30.5
Yes	724	71.3	340	73.0	374	69.5
Employed						
No	431	91.7	120	87.0	308	93.6
Yes	39	8.3	18	13.0	21	6.4
SRH						
Excellent/good/fair	984	97.3	453	98.1	519	96.6
Poor	27	100.0	9	1.9	18	3.4

	**Mean**	**SD**	**Mean**	**SD**	**Mean**	**SD**

Age	43.63	16.48	43.81	16.41	43.50	16.64
Education	3.95	2.03	4.28	2.12	3.63	1.88
Family income	5.36	2.64	5.53	2.63	5.15	2.64
SRH	2.33	1.10	2.22	1.06	2.44	1.12
Discrimination	0.54	1.21	0.59	1.34	0.51	1.09
Distress	8.16	4.72	7.82	4.32	8.44	5.05

*SRH, self-rated health*.

Table 1 also shows descriptive statistics based on gender. While education and income were higher in men than in women, SRH and psychological distress were worse in women than in men. Men reported higher levels of discrimination than women.

### Bivariate Analysis

Table 2 shows bivariate correlations in the pooled sample. As this table shows, discrimination and psychological distress showed a weak, yet positive association (*r* = 0.16). In the pooled sample, psychological distress was also associated with gender, education, income, marital status, and SRH.

**Table 2:** **Summary of bivariate correlations in the pooled sample of Arab Americans**.

	1	2	3	4	5	6	7	8	9	10
1 Gender (male)	1.00	0.01	−0.01	0.16**	0.07*	0.04	0.11*	−0.04	0.03	−0.07*
2 Age			−0.05	−0.04	−0.09**	0.06*	−0.22**	0.09**	−0.19**	−0.04
3 Nativity				0.22**	0.28**	−0.30**	0.26**	−0.03	0.07*	−0.02
4 Education					0.45**	−0.01	0.19**	−0.09**	0.05	−0.08*
5 Family income						0.00	0.19**	−0.15**	−0.01	−0.07*
6 Married							−0.24**	0.00	−0.10**	−0.07*
7 Employment								−0.03	0.10*	0.04
8 SRH (poor)									0.04	0.25**
9 Discrimination										0.16**
10 Psychological distress										1.00

*p < 0.05.

**p < 0.01.

*SRH, self-rated health*.

Table 3 shows bivariate correlations based on gender. As this table shows, discrimination and psychological distress were correlated in males (*r* = 0.22) and females (*r* = 0.10).

**Table 3:** **Summary of bivariate correlations based in Arab American male and females**.

	1	2	3	4	5	6	7	8	9
1 Age	1.00	−0.05	−0.16**	−0.16**	−0.14**	−0.21**	0.09*	−0.21**	−0.05
2 Nativity	−0.05	1.00	0.27**	0.28**	−0.26**	0.28**	−0.05	0.14**	−0.03
3 Education	0.08	0.20**	1.00	0.43**	0.00	0.19**	−0.08	0.04	−0.07
4 Family income	−0.01	0.29**	0.47**	1.00	0.01	0.17**	−0.21**	0.02	−0.09
5 Married	0.30**	−0.36**	−0.03	−0.01	1.00	−0.24**	−0.01	−0.14**	−0.09*
6 Employment	−0.28**	0.22**	0.16	0.26**	−0.21*	1.00	−0.06	0.10	0.08
7 SRH (poor)	0.09	−0.02	−0.10*	−0.07	0.02	0.00	1.00	0.07	0.28**
8 Discrimination	−0.17**	0.00	0.06	−0.03	−0.07	0.10	0.01	1.00	0.22**
9 Psychological Distress	−0.03	−0.01	−0.07	−0.05	−0.02	0.00	0.19**	0.10*	1.00

*p < 0.05.

**p < 0.01.

*SRH, self-rated health; Females, upper diagonal; Males; lower diagonal*.

### Multivariable Analysis

As Table 4 shows, in the pooled sample, discrimination was positively associated with psychological distress (*B* = 0.62, 95% CI = 0.22–1.03, *p* = 0.003). We found a significant gender × discrimination interaction in the pooled sample (*B* = 0.79, 95% CI = 0.01–1.59, *p* = 0.050), suggesting a stronger association in males than in females.

**Table 4:** **Summary of linear regression analysis between discrimination and psychological distress in the pooled sample of Arab Americans**.

	Model 1		Model 2		Model 3		Model 4		Model 5	
	*B* (SE)	95% Confidence interval (CI)	*B* (SE)	95% CI	*B* (SE)	95% CI	*B* (SE)	95% CI	*B* (SE)	95% CI
Age	0.00 (0.01)	−0.03 to 0.02	0.00 (0.01)	−0.03 to 0.03	−0.01 (0.01)	−0.03 to 0.02	0.01 (0.01)	−0.02 to 0.03	0.00 (0.01)	−0.02 to 0.03
Gender (men)	−0.44 (0.59)	−1.60 to 0.73	−0.58 (0.61)	−1.77 to 0.62	−0.78 (0.58)	−1.91 to 0.36	−0.78 (0.57)	−1.91 to 0.34	−0.35 (0.61)	−1.55 to 0.85
Nativity	−1.12 (0.61)	−2.33 to 0.08	−1.35 (0.70)	−2.73 to 0.03	−1.36 (0.67)*	−2.67 to 0.05	−1.30 (0.66)*	−2.60 to 0.01	−1.41 (0.66)*	−2.70 to 0.11
Education	–		−0.02 (0.16)	−0.34 to 0.31	0.03 (0.16)	−0.28 to 0.33	0.01 (0.16)	−0.30 to 0.31	0.01 (0.15)	−0.30 to 0.31
Family income	–		−0.11 (0.11)	−0.32 to 0.11	−0.06 (0.11)	−0.27 to 0.15	−0.06 (0.11)	−0.27 to 0.15	−0.06 (0.10)	−0.26 to 0.15
Married	–		−1.20 (0.63)	−2.43 to 0.04	−1.18 (0.60)*	−2.36 to 0.01	−0.98 (0.59)	−2.15 to 0.19	−1.02 (0.59)	−2.19 to 0.14
Employment	–		0.37 (1.03)	−1.66 to 2.40	0.42 (0.98)	−1.51 to 2.35	0.41 (0.97)	−1.50 to 2.32	0.46 (0.97)	−1.44 to 2.37
SRH (poor)	–				7.27 (1.16)***	4.99–9.55	7.07 (1.15)***	4.81–9.34	7.06 (1.15)**	4.81–9.32
Discrimination	–						0.62 (0.21)**	0.22–1.03	0.16 (0.31)	−0.46 to 0.78
Gender (men) × discrimination	–								0.79 (0.40)***	0.01–1.59
Constant	9.15 (0.73)***	7.71–10.58	10.61 (1.09)***	8.46–12.75	10.06 (1.04)***	8.01–12.11	9.19 (1.07)***	7.09–11.29	9.01 (1.07)***	6.90–11.11

*SRH, self-rated health*.

*p < 0.05, **p < 0.01, ***p < 0.001.

As Table 5 shows, in our gender-specific model, higher discrimination was associated with higher psychological distress among male (*B* = 0.87, 95% CI = 0.33–1.42, *p* = 0.002), but not female (*B* = 0.18, 95% CI = −0.43 to 0.78, *p* = 0.567), Arab Americans.

**Table 5:** **Summary of linear regression analysis between discrimination and psychological distress among male and female Arab Americans**.

	Model 1		Model 2		Model 3		Model 4	
	*B* (SE)	95% Confidence interval (CI)	*B* (SE)	95% CI	*B* (SE)	95% CI	*B* (SE)	95% CI
**Men**								
Age	−0.02 (0.02)	−0.06 to 0.02	−0.03 (0.02)	−0.07 to 0.01	−0.03 (0.02)	−0.07 to 0.01	−0.01 (0.02)	−0.05 to 0.02
Nativity	−0.48 (0.79)	−2.03 to 1.07	−0.52 (0.88)	−2.25 to 1.21	−0.59 (0.84)	−2.25 to 1.07	−0.71 (0.83)	−2.34 to 0.92
Education	–	–	−0.05 (0.22)	−0.49 to 0.38	−0.06 (0.21)	−0.47 to 0.36	−0.08 (0.21)	−0.49 to 0.33
Family income	–	–	−0.19 (0.13)	−0.46 to 0.07	−0.10 (0.13)	−0.36 to 0.16	−0.1 (0.13)	−0.35 to 0.15
Married	–	–	−1.53 (0.83)	−3.16 to 0.10	−1.38 (0.79)	−2.94 to 0.19	−1.02 (0.79)	−2.57 to 0.53
Employment	–	–	0.05 (1.43)	−2.76 to 2.87	0.39 (1.37)	−2.32 to 3.09	0.55 (1.35)	−2.11 to 3.21
SRH (poor)	–	–	–	–	7.57 (1.57)***	4.48–10.66	7.33 (1.54)***	4.29–10.38
Discrimination	–	–	–	–	–	–	0.87 (0.28)**	0.33–1.42
Constant	9.67 (0.91)***	7.88–11.46	12.46 (1.49)***	9.53–15.39	11.55 (1.44)***	8.72–14.38	10.15 (1.48)***	7.24–13.07
**Women**								
Age	0.02 (0.02)	−0.03 to 0.06	0.04 (0.02)	−0.01 to 0.08	0.03 (0.02)	−0.02 to 0.08	0.03 (0.02)	−0.01 to 0.08
Nativity	−2.11 (0.95)*	−4.00 to 0.22	−3.12 (1.16)**	−5.41 to 0.83	−2.98 (1.09)**	−5.14 to 0.82	−2.90 (1.10)**	−5.08 to 0.71
Education	–	–	−0.03 (0.25)	−0.52 to 0.46	0.07 (0.23)	−0.39 to 0.53	0.06 (0.23)	−0.40 to 0.53
Family income	–	–	0.13 (0.20)	−0.27 to 0.53	0.07 (0.19)	−0.31 to 0.44	0.07 (0.19)	−0.31 to 0.44
Married	–	–	−1.81 (1.13)	−4.06 to 0.43	−1.93 (1.07)	−4.04 to 0.19	−1.86 (1.08)	−3.99 to 0.27
Employment	–	–	0.36 (1.50)	−2.6 to 3.33	0.30 (1.41)	−2.49 to 3.10	0.27 (1.41)	−2.54 to 3.07
SRH (poor)	–	–	–	–	6.52 (1.69)***	3.16–9.88	6.46 (1.70)***	3.08–9.83
Discrimination	–	–	–	–	–	–	0.18 (0.31)	−0.43 to 0.78
Constant	8.00 (1.27)***	5.48–10.52	8.08 (1.67)***	4.77–11.38	7.77 (1.57)***	4.66–10.89	7.55 (1.62)***	4.33–10.77

*SRH, self-rated health*.

*p < 0.05, **p < 0.01, ***p < 0.001.

## Discussion

Our study revealed two main findings; first, discrimination was associated with psychological distress among Arab Americans. Second, gender moderates the association between perceived discrimination and psychological distress in the Arab American community, with the association being stronger for males than for females.

Our first finding that discrimination is associated with psychological distress is in line with previous studies (15). Discrimination is also associated with other undesired physical and mental health outcomes (5–9) including but not limited to depression (10), anxiety (11), substance use (12), and suicide (13, 14). Most of the published literature on mental health correlates of discrimination in the United States is, however, limited to Blacks (3, 8, 16–22) and Hispanics (23–25). This study extends the limited literature on Arab Americans (26–28).

Our second finding on gender as a moderator of the association between discrimination and psychological distress is also in line with findings in other ethnic groups. In studies that have mostly enrolled Black Americans, men have reported higher rates of perceived discrimination compared with women (41, 43, 44). Building on multiracial feminist theories, Harnois and Ifatunji (59) offered a theoretical framework to understand interpersonal racial discrimination as gendered measures, gendered models, and gendered phenomena. The intersectional framework that Harnois and Ifatunji proposed suggests that, while there are some discriminatory practices that are directed at both male and female minorities, some forms of discrimination will affect men more than women, some will affect women more than men, and some forms may be gender-specific (59). For instance, due to controlling images that are gendered, among Blacks, men are content with stereotypes such as the lower-class, hyper-sexual “thug,” while women face stereotypes of mammies, matriarchs, jezebels, and welfare queens (60–63). There is evidence suggesting that some racial stereotypes are indeed gender-specific (64, 65). Men of color may be more commonly discriminated against when they look for a job; as in some racial groups, women may have better job opportunities than men (66).

Multiple hypotheses have been proposed to explain the gender gap in perceived discrimination among minorities. The subordinate male target hypothesis argues that minority men are subject to more experiences of discrimination (41). The race–gender intersectionality hypothesis also argues that it is neither race nor gender, but their intersection that shapes experiences such as discrimination (41, 59). According to recent work by Ifatunji and Harnois on data from the National Survey of American Life as well as the Detroit Area Study, everyday discrimination could justify the subordinate male hypothesis, while major life discrimination could better fit with the intersectionality hypothesis (41). In the case of Blacks, more negative attitudes toward Black males compared with Black females as a result of stereotypes that exist around race and gender may be due to the attribution of aggression and anti-intellectuality to Black males (40, 42, 67, 68). It has been shown that in schools, Black boys more commonly receive negative treatment compared to Black girls (42, 69–73). This may be in part due to differences in attending to threat, perceived threat, and stereotype threat for Black men that may also be in part reinforced by their body size, appearance, or behaviors (74–76). Such differences can be seen in neuroimaging studies (77) or neurocognitive tests (78). Such threat attention may also have implications in interactions between minority men and police (79). In the case of Arab Americans, with the same reasoning, men may be more frequently exposed to discrimination due to stereotype threat.

Among minorities, gender may shape the frequency of messages around race, racism, discrimination, immigration, and religion in families. For instance, among Black families, boys and girls receive different levels of parental messages considering discrimination and racial barriers (80, 81). Differential messages that are being communicated to boys and girls may cause differential awareness or attention toward factors such as discrimination and associated cues in one gender. Such practices may influence perception as well as impact of discrimination between males and females. Another factor may be masculine role norms that may moderate the link between discrimination and psychological distress in minorities (10, 82). Yet another explanation is restricted emotion expression in men in general, and in men of color in particular. Men of color who live under oppression learn to have a higher self-reliance that may increase psychological distress (82). Sex and gender role, self-image, peer appraisal, gender identity, and access to social support may also have a differential role for male and females (42). In addition, males and females use different coping styles to deal with stressors, including discrimination (42, 83). Overall, the interaction of gender and culture/ethnicity should be considered when studying the mental health of minority individuals (42).

Our findings should be interpreted in the context of Arab American culture that shapes traditional gender roles, norms, and identities. In many Arab and Muslim countries, gender roles are very distinct for men and women. Major decisions are made by men, as men are very authoritative toward women, who are more submissive. Men are the main breadwinners and are involved with the labor market, so they get more involved with the society in general. Instead, women’s social lives are more limited, as they spend most of their time inside with their children and close relatives. Men endorse high levels of hegemonic masculinities and are not expected to share their emotions. As a result, men internalize their emotional problems, do not seek emotional social support, and do not communicate about their problems. This increases vulnerability of men compared to women, who can easily seek emotional support and share and communicate about their emotional problems and stressors (84–89).

Research has shown that in addition to distress, discrimination is also associated with drug use; an effect which seems to be stronger for males than for females (12). Brondolo et al. (90) showed that discrimination may differently influence substance use of men and women, with stronger effects in men compared with women. Our study, however, did not explore gender differences in the effects of discrimination on substance use in the Arab community.

In contrast to our study, which did not find an association between discrimination and distress among females, other studies have found such association between experiences of discrimination and poor mental health in female minorities (91). Coping, however, may explain some gender differences in the consequences of discrimination. Among Blacks, women have a higher tendency to use avoidant coping mechanisms in response to race-related stress, rather than recruiting more combative forms of coping, such as using one’s voice as power (83). Avoidant coping may mediate the association of gendered racism with heightened distress (83).

Gender may mitigate how exposure to the same discriminatory experience is perceived as stressful (21). Gender influences the decision to seek emotional support following exposure to discrimination and other stressors, with women having a higher tendency to seek social support than men; this support, sought from a wide range of sources including family and friends, is used as a coping strategy against racism (92–95), which is in line with the notion that women are better users of social support in general than are men (96). Family members and friends may also be differently available as a source of social support for men and women (10).

Our findings indicating gender differences in the psychological correlates of perceived discrimination have implications for public health and clinical practice with the Arab American community. Perceived discrimination may be a more salient contributor to poor mental health for male compared to female Arab Americans. Asking about perceived discrimination may be an important aspect of mental health screening both in community and clinical settings and may be an important step toward the diagnosis and treatment of psychopathology for Arab Americans, particularly men.

Our findings may also have implications for future research on the intersection of race and ethnicity, discrimination, and health. Further research is needed to better understand mechanisms by which male and female Arab Americans respond differently to similar discrimination experiences. Differential coping mechanisms may be a hypothetical explanation for worse effects of discrimination on the mental health of male minorities.

This study is subject to a few limitations. First, we measured psychological distress rather than clinical diagnosis of psychiatric disorders. Although we used a valid measure of psychological symptoms, structural interviews could have provided more accurate results. Second, we did not account for a previous diagnosis of psychopathology, or the use of anti depressant or anxiolytic medications which may interfere with the psychological symptoms. Third, we did not account for discrimination due to other factors such as gender and age. Although previous research exists on the link between discrimination and the mental health outcomes of Arab Americans, the current study was one of the first attempts to understand gender differences in these links. Using a representative sample of Arab Americans is also a point of strength in this study.

In conclusion, the current study showed that there is an association between perceived discrimination and psychological distress among Arab Americans. This study also showed major gender differences in this link, with stronger association displayed among men than women. Given the lack of research on processes and of mental health in Arab American communities, further investigation is needed to study how sociodemographic factors, identity, discrimination, acculturation, access to health care, and coping shape the psychopathology of this demographic group. Such findings have clinical and public health implications for the mental health promotion of Arab American communities.

## Author Contributions

The original idea of this analysis was developed by SA; SA also analyzed the data. ML drafted the manuscript. SA and ML approved the final version of the manuscript.

## Conflict of Interest Statement

The authors declare that the research was conducted in the absence of any commercial or financial relationships that could be construed as a potential conflict of interest.
